# Evaluation of increasing standardized ileal digestible valine:lysine ratio on growth performance and plasma urea nitrogen of 21 to 116 kg pigs

**DOI:** 10.1093/tas/txag079

**Published:** 2026-06-15

**Authors:** Samantha A Swanson, Andrew W Boschert, Mikayla S Spinler, Grady A Privett, Robert D Goodband, Mike D Tokach, Jason C Woodworth, Joel M DeRouchey, Katelyn N Gaffield, Jordan T Gebhardt

**Affiliations:** Department of Animal Sciences and Industry, College of Agriculture, Kansas State University, Manhattan, KS 66506-0201, United States; Department of Animal Sciences and Industry, College of Agriculture, Kansas State University, Manhattan, KS 66506-0201, United States; Department of Animal Sciences and Industry, College of Agriculture, Kansas State University, Manhattan, KS 66506-0201, United States; Department of Animal Sciences and Industry, College of Agriculture, Kansas State University, Manhattan, KS 66506-0201, United States; Department of Animal Sciences and Industry, College of Agriculture, Kansas State University, Manhattan, KS 66506-0201, United States; Department of Animal Sciences and Industry, College of Agriculture, Kansas State University, Manhattan, KS 66506-0201, United States; Department of Animal Sciences and Industry, College of Agriculture, Kansas State University, Manhattan, KS 66506-0201, United States; Department of Animal Sciences and Industry, College of Agriculture, Kansas State University, Manhattan, KS 66506-0201, United States; Department of Animal Sciences and Industry, College of Agriculture, Kansas State University, Manhattan, KS 66506-0201, United States; Department of Diagnostic Medicine/Pathobiology, College of Veterinary Medicine, Kansas State University, Manhattan, KS 66506-0201, United States

**Keywords:** finishing pig, lysine, valine

## Abstract

Three experiments evaluated the effects of increasing standardized ileal digestible (SID) Val:Lys ratio on growth performance and plasma urea nitrogen (PUN) of pigs from 21 to 116 kg. In Exp. 1, 351 pigs (initially 21.4 ± 0.35 kg) were used in a 21-d study. Pens of pigs were blocked by body weight (BW) and randomly allotted to one of six dietary treatments with five pigs per pen and 12 replications per treatment. The SID Val levels were 60, 63, 66, 69, 72, and 75% of Lys. Pigs were weighed on d 0 and 21 to determine average daily gain (ADG), average daily feed intake (ADFI) and gain:feed ratio (G:F). A blood sample was collected from 2 pigs per pen on d 10 after a 5-h fasting period. Gain:feed ratio increased (linear, *P = *0.012) as SID Val:Lys ratio increased, but with little improvement past 69% SID Val:Lys ratio as supported by broken-line analysis. Increasing Val:Lys ratio increased (linear, *P = *0.029) PUN but no differences were observed for ADG or ADFI. In Exp. 2, 647 pigs (initially 38.6 ± 0.90 kg) were used in a 27-d experiment. Pens of 8 or 9 pigs were blocked by BW and randomly allotted to one of six dietary treatments with 12 replications per treatment. The SID Val levels were 60, 63, 66, 69, 72, and 75% of Lys. Pigs were weighed on d 0 and 27 and after a 10 h fasting period on d 15, blood was collected from 3 pigs per pen. Final BW, ADG, and ADFI increased (quadratic, *P *< 0.05) as SID Val:Lys ratio increased. A quadratic polynomial analysis indicated a SID Val:Lys ratio of near 69% maximized ADG. As the SID Val:Lys ratio increased, both G:F and PUN decreased (linear, *P *< 0.050). After a 30-d common feeding period, the same 647 pigs (initially 90.0 ± 1.86 kg) from Exp. 2 were used in another 27-d experiment (Exp. 3). Pens of pigs were randomly reallotted to one of six dietary treatments and weighed on d 0 and 27. The SID Val levels were 63, 66, 69, 72, 75, and 78% of Lys. On d 15, after a 10-h fasting period, blood was collected from 3 pigs per pen. Average daily gain and ADFI increased (linear, *P *< 0.05) as SID Val:Lys ratio increased. However, little improvement in ADG was observed when feeding greater than 69% SID Val: Lys ratio. Final BW also tended to increase (linear, *P = *0.078) as SID Val:Lys ratio increased; however, there was no evidence of an effect on G:F or PUN. These results indicate a SID Val:Lys ratio of 69% appears to maximize ADG or G:F for 21 to 40 and 38 to 67 kg pigs. In 90 to 116 kg pigs, a ratio of at least 69% SID Val:Lys appeared to be sufficient for growth performance.

## Teaser

These results indicate a standardized ileal digestible valine:lysine ratio of at least 69% appears to maximize gain or feed efficiency for 21 to 116 kg pigs.

## Introduction

Amino acid (AA) requirement estimates for growing–finishing pigs are commonly expressed relative to lysine on a standardized ileal digestible (SID) basis. This is because Lys is typically the first limiting AA in corn–soybean meal-based diets. Within corn-soybean meal diets, Val is recognized as the fifth limiting AA after Lys, Met, Thr, and Trp (Figueroa et al. 2003). Valine is a branched-chain amino acid (BCAA) that plays important roles in protein synthesis, muscle accretion, immune function, and gastrointestinal integrity (Wang et al. 2023). Historically, there has been limited research establishing Val requirement estimates across the growing-finishing period. The NRC (2012) estimates SID Val:Lys ratios between 63.4% and 67.2% for pigs from 11 to 135 kg BW. However, at the time of publication, no studies had evaluated Val requirements in pigs with an average BW greater than 27 kg.

Since the publication of NRC (2012), several studies have evaluated Val requirement estimates in pigs greater than 27 kg, although results are variable. Previous studies contain a variety of experimental methodologies, statistical models, and diet compositions. Gonçalves et al. (2018) observed that pigs weighing 25 to 45 kg required an SID Val:Lys ratio of 73% to maximize ADG, although a ratio of 68% achieved 99% of maximal ADG response and 69% fully maximized G:F. More recently, Wu et al. (2024) estimated SID Val:Lys requirements of 62 to 68% for pigs weighing 40 to 75 kg and 64 to 71% for pigs weighing 75 to 100 kg, depending on the statistical model used. Few studies have evaluated Val requirement estimates across the entire growing-finishing period. Therefore, the objective of this project was to determine the SID Val:Lys ratio requirement estimate for 21 to 116 kg pigs in 3 experiments.

## Materials and methods

The Kansas State University Institutional Animal Care and Use Committee approved the protocol used in these experiments (4942, 4485.42, and 4485.5).

### General

These experiments were conducted at the Kansas State University Swine Teaching and Research Center in Manhattan, KS. The pigs originated from a porcine reproductive and respiratory syndrome- and *Mycoplasma hyopneumoniae*-free herd. In all experiments, the barns were enclosed and environmentally regulated. In Exp. 1, each pen (1.52 × 1.52 m) provided 0.46 m^2^ per pig and contained a 4-hole stainless steel dry self-feeder (Thorp Equipment Inc., Thorp, WI) and nipple waterer to provide ad libitum access to feed and water. In Exp. 2 and 3, each pen (2.44 m × 3.05 m) provided 0.74 m^2^ per pig and was equipped with a 2-hole dry, single-sided feeder (Farmweld, Teutopolis, IL) and 1-cup waterer to provide ad libitum access to feed and water. Pens in Exp. 1 had tri-bar metal flooring while pens in Exp. 2 and 3 had completely slatted concrete flooring with a 1.21 m pit underneath for manure storage. In each experiment, each weight block had a similar number of barrows and gilts.

Treatment diets for Exp. 1 were manufactured at the O. H. Kruse Feed Technology Innovation Center (Table 1; Manhattan, KS). Experiment 2 and 3 diets were manufactured at Hubbard Feeds (Table 1; Beloit, KS). In Exp. 1 and 2, dietary treatments contained SID Val:Lys ratios of 60, 63, 66, 69, 72, and 75%. In Exp. 3, dietary treatments contained SID Val:Lys ratios of 63, 66, 69, 72, 75, and 78%. In each experiment, a basal diet was formulated with the lowest Val:Lys ratio, and L-Val was added to create a diet with the highest Val:Lys ratio. Intermediate SID Val:Lys ratio diets were created by blending the high and low diets. In Exp. 1, daily feed additions were performed manually. In Exp. 2 and 3, daily feed additions to each pen were accomplished using a robotic feeding system (FeedPro, Feedlogic Corp., Wilmar, MN) able to record feed amounts for individual pens. All experimental diets were corn-soybean meal-based and fed in meal form. Dietary nutrient composition and SID AA coefficients were derived from NRC (2012).

**Table 1.** Diet composition (as-fed basis)

		Exp. 1^a^			Exp. 2^b^			Exp. 3^c^	
Item	SID Val:Lys, %^d^:	60		75	60	75		63	78
**Ingredient, %**									
**Corn**		74.28		74.10	81.90	81.75		90.20	90.05
**Soybean meal, 47.73% CP**		22.18		22.19	14.65	14.65		6.65	6.65
**Calcium carbonate**		0.90		0.90	0.90	0.90		0.75	0.75
**Monocalcium P, 21% P**		0.75		0.75	0.85	0.85		0.90	0.90
**Salt**		0.50		0.50	0.50	0.50		0.50	0.50
**L-Lys-HCl**		0.48		0.48	0.46	0.46		0.39	0.39
**DL-Met**		0.17		0.17	0.12	0.12		0.04	0.04
**L-Thr**		0.21		0.21	0.18	0.18		0.14	0.14
**L-Trp**		0.05		0.05	0.05	0.05		0.04	0.04
**L-Ile**		0.07		0.07	0.07	0.07		0.06	0.06
**L-Val**		–		0.17	–	0.15		–	0.15
**Vitamin premix^e^**		0.20		0.20	0.15	0.15		0.15	0.15
**Trace mineral premix^f^**		0.15		0.15	0.15	0.15		0.15	0.15
**Phytase^g^**		0.06		0.06	–	–		–	–
**Standardized ileal digestible amino acids, %**									
**Lys**		1.10		1.10	0.90	0.90		0.65	0.65
**Ile:Lys**		60		60	60	60		60	60
**Leu:Lys**		121		121	128	128		149	149
**Met:Lys**		37		37	36	36		33	33
**Met and Cys:Lys**		59		59	60	60		60	60
**Thr:Lys**		65		65	65	65		67	67
**Trp:Lys**		19.4		19.4	19.0	19.0		18.9	18.9
**Val:Lys**		60		75	60	75		63	78
**His:Lys**		37		37	37	37		39	39
**NE, kcal/kg**		2,482		2,485	2,612	2,615		2,612	2,615
**CP, %**		17.4		17.4	14.4	14.5		11.2	11.2
**Ca, %**		0.63		0.63	0.61	0.61		0.54	0.54
**STTD P, %^h^**		0.40		0.40	0.39	0.39		0.36	0.36
**Ca:P**		1.24		1.24	1.22	1.22		1.13	1.13

a Diets were fed from 21.4 to 38.8 kg BW. The 2 diets were blended to create intermediate treatment diets containing 60, 63, 66, 69, 72, and 75% SID Val:Lys ratio.

b Diets were fed from 38.6 to 66.3 kg BW. The 2 diets were blended to create intermediate treatment diets containing 60, 63, 66, 69, 72, and 75% SID Val:Lys ratio.

c Diets were fed from 90.0 to 116.1 kg BW. The 2 diets were blended to create intermediate treatment diets containing 63, 66, 69, 72, 75 and 78% SID Val:Lys ratio.

d Standardized ileal digestibility (SID) Val:Lys ratio.

e Provided per kg of premix: 1,653,465 IU of vitamin A as retinyl acetate; 661,387 IU of vitamin D_3_ as cholecalciferol; 17,637 IU vitamin E as dl-α-tocopherol acetate; 1,323 mg of vitamin K as menadione; 13.23 mg of vitamin B_12_ as cyanocobalamin; 19,800 mg of niacin; 11,023 mg of pantothenic acid as d-calcium pantothenate; 3,307 mg of riboflavin.

f Provided per kg of premix: 11,000 mg of Cu as copper sulfate; 198 mg I as ethylenediamine dihydriodide; 73,413 mg Fe as ferrous sulfate; 22,046 mg Mn as manganese oxide; 198 mg Se as sodium selenite; 73,413 mg of Zn as zinc sulfate.

g Exp. 1, HiPhorius 2,400 (DSM-Firmenich, Maastricht, Netherlands) was added at 1,500 FYT/kg of feed and provided an estimated release of 0.12% STTD P. Exp. 2 and 3, phytase was included in the vitamin premix at 499,898 FTU/kg of premix and provided an estimated release of 0.12% STTD P.

h Standardized total tract digestible phosphorus.

For all experiments, Val was estimated to be the second-limiting AA, with SID Lys formulated to be 1.10% in Exp. 1, 0.90% in Exp. 2, and 0.65% in Exp. 3. In amino acid ratio studies, dietary Lys must be below the pig’s requirement to accurately determine the requirement for the test amino acid relative to Lys. The projected Lys requirement for pigs within this facility for Exp. 1, 2, and 3 was calculated to be 1.41% (20.4 g/d SID Lys intake), 1.12% (24.6 g/d SID Lys intake), and 0.71% (21.8 g/d SID Lys intake), respectively (Royall et al. 2022). All other AA ratios relative to Lys were maintained above requirement estimates (NRC 2012).

### Experiment 1

A total of 351 pigs (DNA 600 × 241; initially 21.4 ± 0.35 kg) were used in a 21-d experiment. Pens of pigs were blocked by body weight and randomly allotted to 1 of 6 dietary treatments in a randomized complete block design (RCBD) with 5 pigs per pen and 12 replications per treatment. Pigs were weighed on d 0, 10, and 21 for ADG, ADFI and G:F ratio. After a 6-h fasting period on d 10, a blood sample was collected from the jugular vein of 2 pigs per pen (1 barrow and 1 gilt). Whole blood was centrifuged, and plasma was collected and stored at -20°C until analysis. Plasma was analyzed for urea N concentration using a Urea Nitrogen Colorimetric Detection Kit (Arbor Assays, Ann Arbor, MI). SID Valine and SID Lys intake per day was calculated using the overall ADFI and the SID Val and SID Lys concentration of the diets.

### Experiment 2 and 3

A total of 647 pigs (DNA 600 × 241; Exp. 2, initially 38.6 ± 0.90 kg; Exp. 3 initially 90.0 ± 1.86 kg) were used in two 27-d experiments. Pens of 8 or 9 pigs were blocked by BW and randomly allotted to 1 of 6 dietary treatments in a RCBD with 12 replications per treatment. Pigs were weighed on d 0, 14, and 27 for ADG, ADFI, and G:F. After a 10 h fasting period on d 15, 10 mL of blood was collected from the jugular vein of 3 pigs per pen (2 barrows and 1 gilt). Whole blood was centrifuged, and plasma was collected and stored at -20°C until analysis as described for Exp. 1. SID Valine and SID Lys intake per day was calculated as described for Exp. 1. Following the conclusion of Exp. 2, all pigs were fed a common diet for 30 d and then pens were randomly re-allotted to dietary treatments for Exp. 3.

### Chemical analysis

In all experiments, diet samples were collected. In Exp. 1, samples were taken from every 3^rd^ bag during the diet manufacturing process. In Exp. 2 and 3, feed samples were collected from each feeder after the beginning of each trial. Diet samples were stored at −20°C until they were homogenized, subsampled, and submitted for analysis. The high and low diets that were used for blending in all experiments were sent to the University of Missouri-Columbia Agricultural Experiment Station Chemical Laboratories (Columbia, MO) for the determination of amino acids (AOAC 982.30 E[a, b, c] and 988.15), moisture (AOAC 934.01), and ash (AOAC 942.05). Crude protein determination was performed in duplicate using the Dumas combustion method (AOAC 992.15) with a LECO Tru-Mac N Protein analyzer (LECO Corporation, St. Joseph, MI) at Kansas State University on low and high basal diets as well as all blended intermediate diets (Table 2).

**Table 2.** Analyzed nutrient composition of experimental diet (as-fed basis)^a^

		Exp. 1^b^		Exp. 2^c^		Exp. 3^d^	
Item, %	SID Val:Lys, %^e^:	60	75	60	75	63	78
**DM^f^**		87.72	87.84	88.16	87.28	88.01	88.20
**CP^g^**		17.28	16.84	13.68	14.01	11.06	11.01
**Ash**		3.71	4.15	3.57	3.53	3.16	3.07
**Amino acids**							
**Lys**		1.15	1.16	1.02	0.98	0.80	0.84
**Ile**		0.78	0.76	0.64	0.61	0.48	0.48
**Leu**		1.54	1.46	1.28	1.27	1.12	1.10
**Met**		0.37	0.39	0.35	0.32	0.22	0.23
**Cys**		0.31	0.30	0.26	0.26	0.21	0.22
**Thr**		0.74	0.75	0.66	0.67	0.51	0.50
**Trp**		0.21	0.21	0.17	0.17	0.14	0.16
**Val**		0.83	0.90	0.64	0.79	0.51	0.60
**His**		0.46	0.43	0.36	0.35	0.29	0.29

a Diet samples were taken directly after diet manufacturing and stored at −20°C. Values are reported on a total analyzed basis. Composite samples were submitted to University of Missouri-Columbia Agricultural Experiment Station Chemical Laboratories (Columbia, MO) for dry matter, ash and amino acid analysis. Crude protein analysis was performed at Kansas State University.

b Diets were fed from 21.4 to 38.8 kg BW. The 2 diets were blended to create intermediate treatment diets containing 60, 63, 66, 69, 72, and 75% standardized ileal digestibility (SID) Val:Lys ratio.

c Diets were fed from 38.6 to 66.3 kg BW. The 2 diets were blended to create intermediate treatment diets containing 60, 63, 66, 69, 72, and 75% SID Val:Lys ratio.

d Diets were fed from 90.0 to 116.1 kg BW. The 2 diets were blended to create intermediate treatment diets containing 63, 66, 69, 72, 75 and 78% SID Val:Lys ratio.

e Standardized ileal digestibility (SID) Val:Lys ratio.

f DM = dry matter.

g CP = crude protein.

### Statistical analysis

Data were analyzed as a RCBD for a one-way ANOVA using R Studio version 4.3.1 (R Core Team, Vienna, Austria) with pen as the experimental unit. In Exp. 1, BW on d 0 was used as a covariate for analysis on all reported growth performance measures except for d 0 BW. In all experiments, linear and quadratic contrasts were used to evaluate the effects of increasing the SID Val:Lys ratio. For the analysis of plasma urea nitrogen (PUN), treatment was included as a fixed effect, and block and plate were included as random effects. Results from all experiments were considered significant at *P *< 0.05 and tendencies at *P *≤ 0.10. Dose response curves were evaluated using the NLMIXED procedure within SAS (v. 9.4, SAS Institute, Inc., Cary, NC) using linear, quadratic polynomial (QP), and broken-line linear (BBL) models. The best-fitting model was selected using the Bayesian Information Criterion (BIC).

## Results

Results of laboratory analysis indicated nutrient profiles were consistent with expected formulated values (Table 2).

### Experiment 1 (21 to 40 kg)

**Figure 1:**
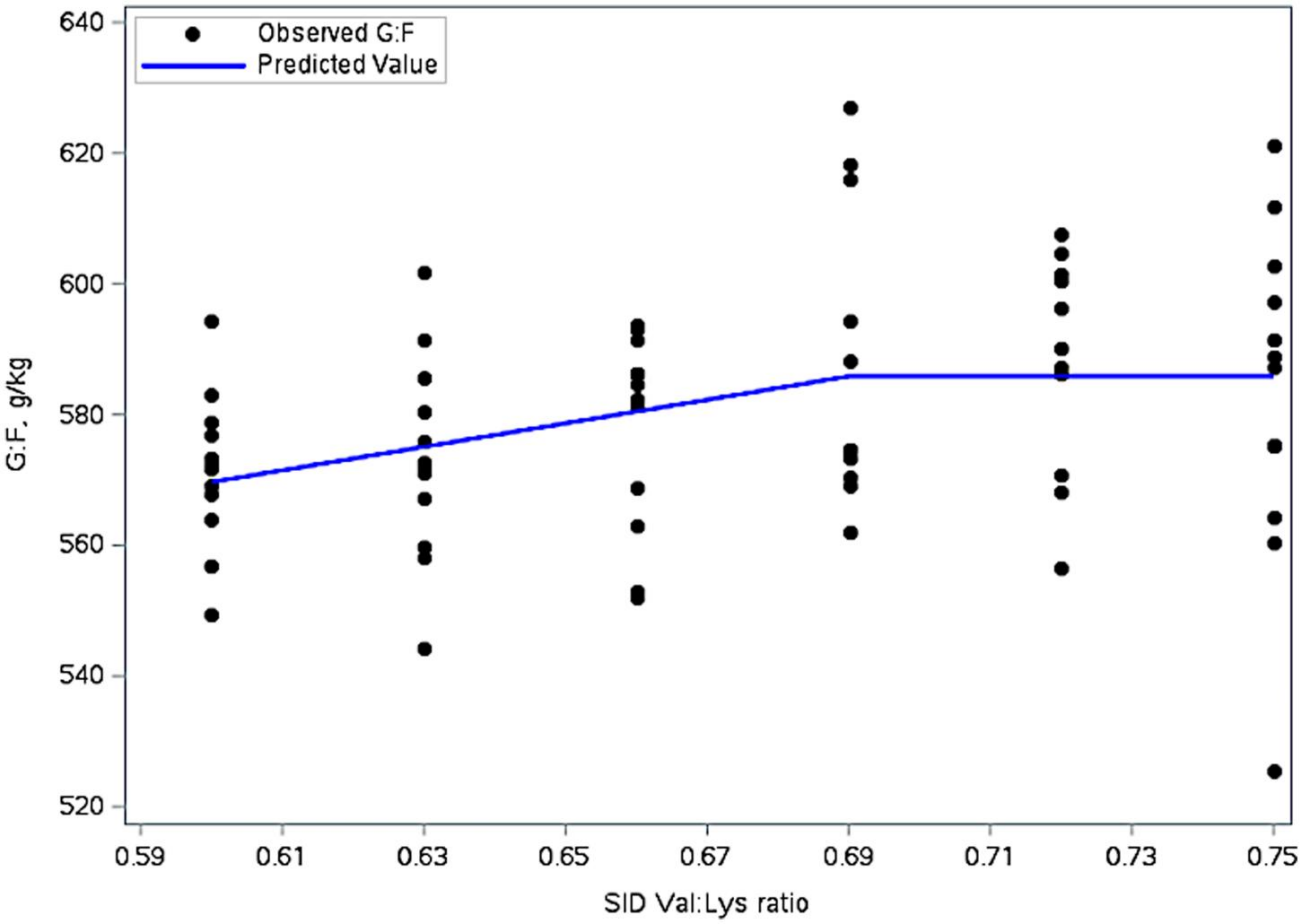
Estimation of standardized ileal digestibility (SID) Val: Lys ratio requirement to maximize G:F of 21- to 40-kg pigs, Exp. 1. A total of 351 pigs (DNA 600 × 241) were used in a 21-d trial. The broken-line linear model (BLL) resulted in the best fit based on the Bayesian Information Criterion. The BLL model predicted no further improvement beyond 69% SID Val:Lys ratio.

Gain: feed ratio increased (linear, *P = *0.012; Table 3) as SID Val:Lys ratio increased, but with little additional improvement beyond 69% SID Val:Lys ratio. Broken-line analysis indicated overall G:F increased with increasing SID Val:Lys ratio, with a breakpoint observed at a ratio of 69% of Lys (Fig. 1). The estimated mean minimum SID Val:Lys ratio required to achieve 99% of the maximum G:F was approximately 66% (Table 4). No other significant differences in growth performance were observed. As the SID Val:Lys ratio increased, there was an increase in PUN concentration (linear, *P = *0.029). Valine intake (g/d and g/kg of gain) increased (linear, *P < *0.001) as the SID Val:Lys ratio increased, while Lys intake g/d was similar across treatments. Lysine intake g/kg of gain decreased (linear, *P = *0.029) as the SID Val:Lys ratio increased.

**Table 3.** Effects of increasing standardized ileal digestible (SID) Val:Lys ratio on growth performance and plasma urea nitrogen of 21- to 40-kg pigs (Exp. 1)^a^

	SID Val:Lys ratio, %							*P =*	
Item	60	63	66	69	72	75	SEM	Linear	Quadratic
**BW, kg**									
**d 0**	21.5	21.5	21.3	21.2	21.6	21.2	0.35	0.380	0.832
**d 21**	39.8	39.7	39.7	39.9	40.0	40.2	0.31	0.162	0.481
**Growth performance**									
**ADG, g**	920	916	921	927	931	936	13.9	0.242	0.739
**ADFI, kg**	1.61	1.60	1.60	1.58	1.58	1.61	0.025	0.815	0.346
**G:F, g/kg**	572	574	577	588	589	582	5.0	0.012	0.252
**Plasma urea N, mg/dL^b^**	5.12	5.56	5.37	5.45	6.48	6.05	0.400	0.029	0.872
**Val intake, g/d**	10.61	11.07	11.59	12.00	12.52	13.29	0.194	< 0.001	0.376
**Val intake, g/kg gain**	11.53	12.08	12.59	12.93	13.45	14.20	0.114	< 0.001	0.301
**Lys intake, g/d**	17.69	17.57	17.56	17.40	17.38	17.73	0.279	0.815	0.346
**Lys intake, g/kg gain**	19.22	19.18	19.07	18.74	18.67	18.94	0.163	0.016	0.243

a A total of 351 pigs (DNA 600 × 241) were used in a 21-d trial with 5 pigs per pen and 12 replications per treatment.

b After a 6-h fasting period, blood samples were taken on d 10 from 2 pigs per pen (1 barrow and 1 gilt).

**Table 4.** Estimated mean lowest standardized ileal digestible (SID) Val:Lys ratio for selected target mean performance levels

	Percent of maximum performance, %			
Item	95%	98%	99%	100%
**Exp. 1, G:F^a^**	52.7	62.5	65.7	69.0
**Exp. 2, ADG^b^**	59.7	63.0	64.7	68.7
**Exp. 3, ADG^c^**	61.4	71.4	74.7	78.0

a Broken line linear question for predicted G:F, g/kg = 585.9 + 179 (SID Val:Lys ratio—0.69) if SID Val:Lys ratio < 0.69, G:F = 585.9 if SID Val:Lys ratio ≥ 0.69.

b QP equation for predicted ADG, g = −6,323.89 × (SID Val:Lys ratio)^2^ + 8,689.46 × (SID Val:Lys ratio) – 1965.16.

c Linear equation for predicted ADG, g = 729.11 + 286.92 × (SID Val:Lys ratio).

### Experiment 2 (38 to 67 kg)

**Figure 2:**
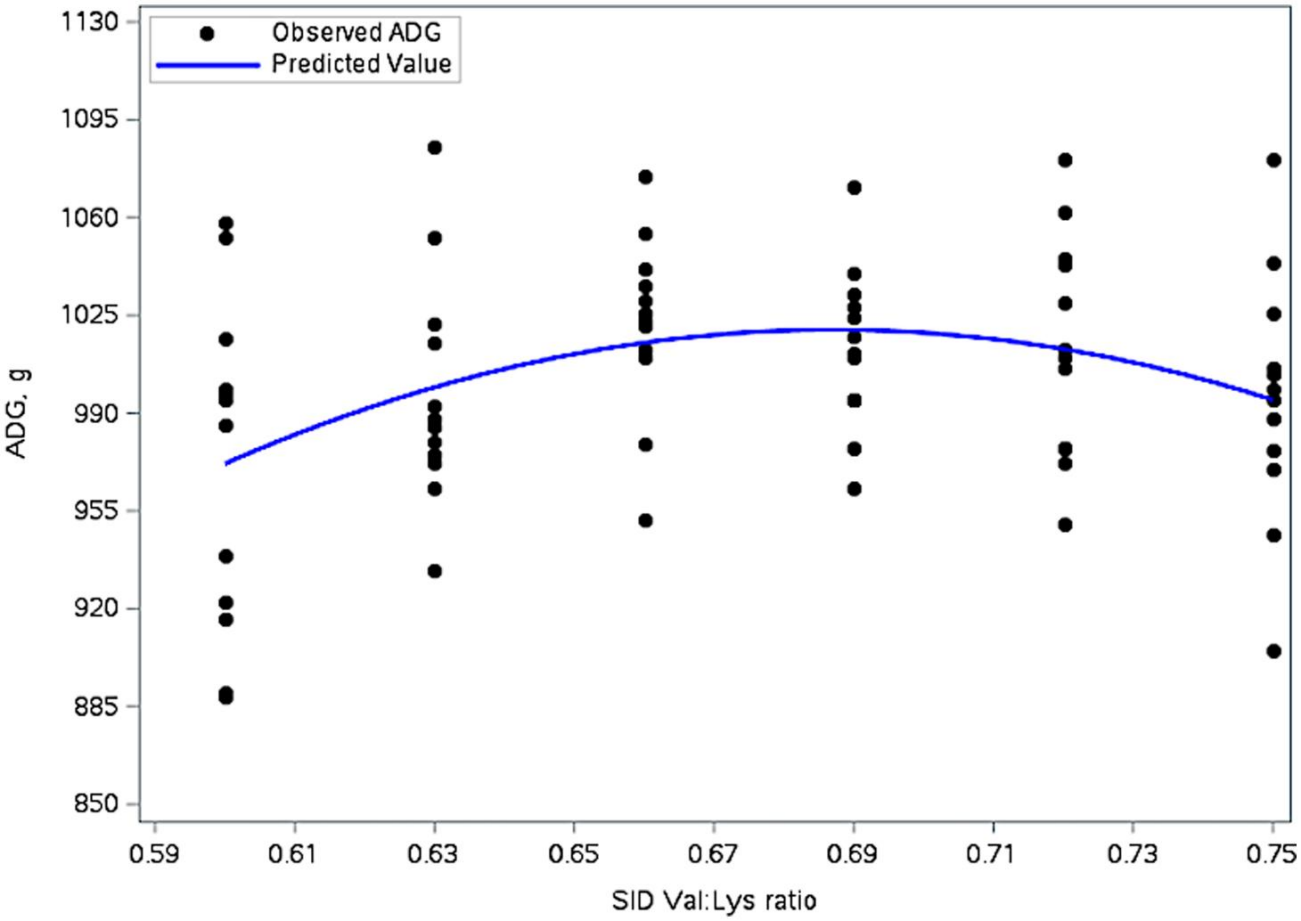
Estimation of standardized ileal digestibility (SID) Val: Lys ratio requirement to maximize ADG of 38- to 67-kg pigs, Exp. 2. A total of 647 pigs (DNA 600 × 241) were used in a 27-d trial. The quadratic polynomial model resulted in the best fit based on the Bayesian Information Criterion. ADG, g = −6,323.89 × (SID Val:Lys ratio)^2^ + 8,689.46 × (SID Val:Lys ratio) – 1965.16. Maximum ADG was calculated to be 68.7% SID Val:Lys.

Final BW, ADG, and ADFI increased (quadratic, *P *< 0.05) as the SID Val:Lys ratio increased (Table 5). Pigs fed the 66% SID Val:Lys ratio had the greatest final BW and ADG, whereas pigs fed 72% SID Val:Lys ratio had the greatest ADFI. A QP analysis indicated ADG was maximized near 69% SID Val:Lys (Fig. 2), but 99% of maximum ADG was near 65% SID Val:Lys ratio (Table 4). However, as the SID Val:Lys increased, G:F decreased (linear, *P *< 0.001), primarily driven by the middle treatments of 63, 66, 69, and 72% SID Val:Lys. Plasma urea nitrogen decreased (linear, *P *= 0.048) with increasing SID Val:Lys ratio. Following the trend observed in ADFI, Val and Lys intake g/d increased (quadratic, *P *< 0.05) with increasing SID Val:Lys ratio. As the SID Val:Lys ratio increased, Val and Lys intake g/kg of gain increased (linear, *P *< 0.05).

**Table 5.** Effects of increasing standardized ileal digestible (SID) Val: Lys ratio on growth performance and plasma urea nitrogen of 38- to 67-kg pigs (Exp. 2)^a^

	SID Val:Lys ratio, %							*P =*	
Item	60	63	66	69	72	75	SEM	Linear	Quadratic
**BW, kg**									
**d 0**	38.6	38.6	38.6	38.6	38.7	38.6	0.90	0.871	0.666
**d 27**	65.3	66.0	66.7	66.5	66.6	66.9	0.83	0.189	0.008
**Growth performance**									
**ADG, g**	972	997	1,022	1,014	1,014	995	12.0	0.118	0.005
**ADFI, kg**	2.09	2.07	2.16	2.20	2.26	2.14	0.023	<0.001	0.002
**G:F, g/kg**	465	482	474	460	449	465	4.1	<0.001	0.721
**Plasma urea N, mg/dL^b^**	8.14	8.16	7.62	7.95	7.16	7.30	1.181	0.048	0.918
**Val intake, g/d**	11.12	11.47	12.53	13.38	14.27	14.06	0.576	<0.001	0.011
**Val intake, kg gain**	11.40	11.50	12.26	13.21	14.10	14.14	0.550	<0.001	0.874
**Lys intake, g/d**	18.14	17.83	18.61	19.02	19.44	18.40	0.960	<0.001	0.007
**Lys intake, kg gain**	18.59	17.87	18.20	18.77	19.20	18.49	0.913	0.008	0.803

a A total of 647 pigs (DNA 600 × 241) were used in a 27-d trial with 8 to 9 pigs per pen and 12 replications per treatment.

b After a 10-h fasting period, blood samples were taken on d 15 from 3 pigs per pen (2 barrows and 1 gilt).

### Experiment 3 (90 to 116 kg)

**Figure 3:**
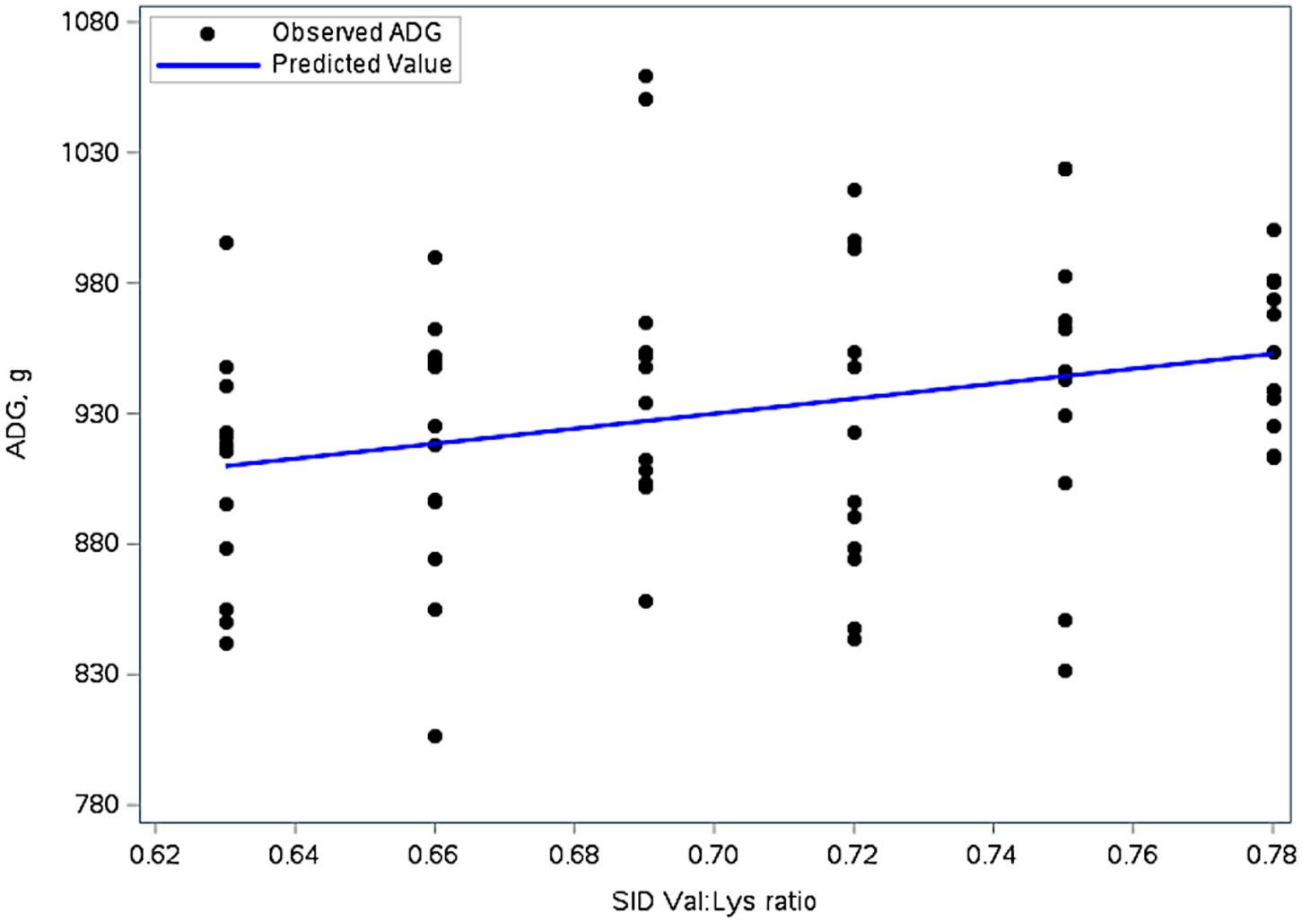
Estimation of standardized ileal digestibility (SID) Val: Lys ratio requirement to maximize ADG of 90- to 116-kg pigs, Exp. 3. A total of 647 pigs (DNA 600 × 241) were used in a 27-d trial. The linear model resulted in the best fit based on the Bayesian Information Criterion. ADG, g = 729.11 + 286.92 × (SID Val:Lys ratio).

Average daily gain and ADFI increased (linear, *P *< 0.05) as the SID Val:Lys ratio increased (Table 6). However, little additional improvement in ADG was observed when SID Val:Lys exceeded 69%. When dose response modeling was performed, the linear model was the best fit (Fig. 3). Final BW also tended to increase (linear, *P = *0.078) as SID Val:Lys ratio increased; however, there was no evidence of an effect on G:F or PUN. Valine intake (g/d and g/kg of gain) and Lys intake g/d increased (linear, *P *< 0.05) as SID Val:Lys ratio increased. There were no differences observed in Lys intake g/kg of gain.

**Table 6.** Effects of increasing standardized ileal digestible (SID) Val:Lys ratio on the growth performance and plasma urea nitrogen of 90- to 116-kg pigs (Exp. 3)^a^

	SID Val:Lys ratio, %							*P =*	
Item	63	66	69	72	75	78	SEM	Linear	Quadratic
**BW, kg**									
**d 0**	89.9	90.0	90.0	90.0	89.9	90.0	1.86	0.975	0.912
**d 27**	115.5	115.6	116.5	116.1	116.2	116.7	2.01	0.078	0.867
**Growth performance**									
**ADG, g**	907	915	946	922	943	956	15.1	0.019	0.910
**ADFI, kg**	2.89	2.90	2.95	3.00	3.05	2.99	0.058	<0.001	0.224
**G:F, g/kg**	314	317	321	309	310	321	5.5	0.956	0.288
**Plasma urea N, mg/dL^b^**	7.95	8.52	8.17	8.19	7.89	9.57	1.014	0.105	0.177
**Val intake, g/d**	13.07	13.55	14.38	15.34	16.19	16.54	0.267	<0.001	0.665
**Val intake, kg gain**	14.41	14.82	15.22	16.67	17.23	17.32	0.231	<0.001	0.587
**Lys intake, g/d**	20.74	20.53	20.84	21.31	21.59	21.21	0.319	<0.001	0.611
**Lys intake, kg gain**	22.88	22.46	22.06	23.15	22.97	22.20	0.321	0.742	0.721

a A total of 647 pigs (DNA 600 × 241) were used in a 27-d trial with 8 to 9 pigs per pen and 12 replications per treatment.

b After a 10-h fasting period, blood samples were taken on d 15 from 3 pigs per pen (2 barrows and 1 gilt).

## Discussion

Valine plays an important role in protein synthesis, muscle accretion, immune function, and gastrointestinal integrity, and the SID Val:Lys ratio is important for optimizing growth performance (Wang et al. 2023). After Lys, Met, Thr, and Trp, Val is generally considered the 5^th^ limiting AA in corn-soybean meal diets (Figueroa et al. 2003). Current NRC (2012) recommendations for SID Val:Lys ratios range from 63.4 to 67.2% for pigs from 11 to 135 kg BW. However, these estimates are largely based on nursery pig data and generated based on models, as the NRC (2012) does not cite any studies evaluating Val requirements in pigs with an average BW above 27 kg. Since the publication of NRC (2012), several studies have evaluated Val requirements. However, reported requirement estimates vary considerably depending on BW range, response criteria, and statistical model used (Liu et al. 2015; Gonçalves et al. 2018; Wu et al. 2024). Results from the present study suggest that NRC (2012) recommendations may underestimate the SID Val:Lys requirement for 21 to 116 kg pigs. However, results are in agreement with other published research reporting optimal SID Val:Lys ratios between 65 and 73% in 20 to 45 kg pigs (Wiltafsky et al. 2009; Waguespack et al. 2012; Gonçalves et al. 2018). In 45 to 130 kg pigs the SID Val:Lys ratio reported in the literature was optimized at a ratio ranging from 62 to 71% (Liu et al. 2015; Clizer et al. 2022; Wu et al. 2024).

In pigs of similar body weight to Exp. 1, Waguespack et al. (2012) and Gonçalves et al. (2018) observed an increase in ADG as the SID Val:Lys ratio increased from 60 to 75% and 59 to 75.5%, respectively. A similar response was not observed in Exp. 1, where there was no evidence of a difference in ADG as SID Val:Lys ratio increased. In Exp. 2, a quadratic polynomial-derived optimum of 69% was observed for ADG. Wu et al. (2024) observed optimal ADG at a SID Val:Lys between 63 and 68% in pigs weighing 40 to 75 kg. Gonçalves et al. (2018) observed similar results to the present study, with 68% SID Val:Lys achieving 99% of maximal ADG. In diets with 30% DDGS, Clizer et al. (2022) reported similar results to the present study where a SID Val:Lys ratio of 68% would yield more than 99% maximum performance for ADG. However, Liu et al. (2015) estimated a wider range, with a SID Val:Lys requirement of 67% using standard broken line or 72% for the QP for ADG in 49 to 70 kg pigs. In heavier pigs with a similar body weight to Exp. 3, previous publications found minimal ADG response when increasing the SID Val:lys ratio (Wu et al. 2024).

The response in ADFI in pigs weighing from 38 to 116 kg was similar to other published data, with greater ADFI as the SID Val:Lys ratio increased (Clark et al. 2017; Gonçalves et al. 2018; Clizer et al. 2022). This may be partially attributed to reduced feed intake observed in pigs fed Val deficient diets (Gloaguen et al. 2011). However, this response was not observed for Exp. 1.

Feed efficiency responses varied across BW phases within these experiments. Gonçalves et al. (2018) observed maximum G:F near 69% SID Val:Lys in pigs of similar BW as used in Exp. 1, which is similar to the response observed in the present study. In contrast to most available literature, G:F decreased as SID Val:Lys increased in 38 to 67 kg pigs, possibly due to disproportionate increases in feed intake relative to ADG. Unlike Exp. 1 and 2, there was a lack of G:F response observed in Exp. 3 similar to that observed by Wu et al. (2024). Wu et al. (2024) theorized that the lack of response was in part attributed to the Leu level in the diet. Similar to the Wu et al. (2024) study, higher dietary Leu concentrations were present in the current study for Exp. 3. The excess Leu may increase the nutritional requirement for Val, as Leu may enhance oxidation and catabolism of BCAA (Wiltafsky et al. 2010). However, the response observed in ADG does not support this, as there was little numerical improvement in ADG after an SID Val:Lys ratio of 69%, whereas Wu et al. (2024) observed 73% SID Val:Lys to have the greatest ADG and BW.

Plasma urea nitrogen reflects the breakdown of excess amino acids and is commonly used as an indicator of nitrogen utilization (Coma et al. 1995; Knowles 1997; Whang and Easter 2000). In Exp. 2, PUN decreased as SID Val:Lys increased, indicating enhanced nitrogen utilization as Val supply approached adequacy. In contrast, PUN increased in Exp. 1 as Val increased, suggesting a potential oversupply of Val at the higher titration levels, particularly at 72 and 75%, where PUN increased 1.03 mg/dL from 69% to 72%. The SID Val:Lys ratio did not appear to impact PUN in Exp. 3, which may be a function of the total Lys intake as described below.

For SID Val:Lys ratio experiments, Lys must be limiting so that changes in Val supply alter growth performance responses. If Lys is supplied in excess, the optimal SID Val:Lys ratio requirement may be underestimated. In the present study, this condition was met as SID Lys intake/kg gain was below predicted requirements for pigs in this facility based on previous research (Royall et al. 2022). In Exp. 1, SID Lys intake ranged from approximately 18.7 to 19.2 g/kg gain compared with a projected requirement of 22.6 g/kg gain, while in Exp. 2, SID Lys intake ranged from 17.9 to 19.2 g/kg gain relative to a predicted requirement of 24.6 g/kg gain. In Exp. 3, SID Lys intake averaged approximately 22 g/kg gain, which is slightly lower than the predicted requirement of 23 g/kg gain for pigs of this body weight (Royall et al. 2022).

In conclusion, results from the present study demonstrate in Exp. 1 a SID Val:Lys ratio near 69% optimizes feed efficiency in pigs weighing 21 to 40 kg. Results from Exp. 2 align closely with existing literature and indicate that a SID Val:Lys ratio of approximately 69% is sufficient to maximize ADG in pigs weighing 38 to 67 kg, which is slightly greater than NRC (2012) estimates. In heavier pigs, Exp. 3 suggests that a SID Val:Lys ratio of at least 69% may support maximal growth, although responses are modest. Collectively, these results indicate a SID Val:Lys ratio of 69% appears to maximize ADG or G:F for 21 to 40 and 38 to 67 kg pigs, with 99% of maximum performance achieved around 65 to 66% SID Val:Lys. In 90 to 116 kg pigs, a ratio of at least 69% SID Val:Lys appeared to be sufficient for growth performance.

## List of abbreviations

AA: amino acid
ADFI: average daily feed intake
ADG: average daily gain
BBL: broken-line linear
BCAA: branched-chain amino acid
BCAT: branched-chain aminotransferase
BCKDH: branched-chain α-keto acid dehydrogenase
BW: body weight
G: F: gain-to-feed
PUN: plasma urea nitrogen
QP: quadratic polynomial
RCBD: randomized complete block design
SID: standardized ileal digestibility
